# Actual Body Weight and the Parent’s Perspective of Child’s Body Weight Among Canadian First Nations Children

**DOI:** 10.3390/children13091197

**Published:** 2026-09-04

**Authors:** Chandima P. Karunanayake, James A. Dosman, Jeremy Seeseequasis, Carol Naytowhow, Reynaldo Lindain, Warren Seesequasis, Kathleen McMullin, Meera J. Kachroo, Shelley Kirychuk, Punam Pahwa

**Affiliations:** 1 Canadian Centre for Rural and Agricultural Health, University of Saskatchewan, 104 Clinic Place, Saskatoon, SK S7N 2Z4, Canada; james.dosman@usask.ca (J.A.D.); jccquasis22@gmail.com (J.S.); kathleen.mcmullin@usask.ca (K.M.); meera.kachroo@usask.ca (M.J.K.); shelley.kirychuk@usask.ca (S.K.); pup165@mail.usask.ca (P.P.); 2 Department of Medicine, College of Medicine, University of Saskatchewan, Royal University Hospital, 103 Hospital Drive, Saskatoon, SK S7N 0W8, Canada; 3 Community A, P.O. Box 250, Montreal Lake, SK S0J 1Y0, Canada; carolnaytowhow@hotmail.com (C.N.); director@wchealth.ca (R.L.); 4 Community B, P.O. Box 96, Duck Lake, SK S0K 1J0, Canada; warrenseese@hotmail.com; 5 Department of Community Health & Epidemiology, College of Medicine, University of Saskatchewan, 107 Wiggins Road, Saskatoon, SK S7N 5E5, Canada

**Keywords:** First Nations, children, indigenous health, body weight, body mass index

## Abstract

**Highlights:**

**What are the main findings?**

- Many First Nations parents had difficulty accurately identifying their child’s weight status, with 42.1% misclassifying their child’s weight category;
- Children who were more physically active were more likely to have their weight status misclassified by their parents.

**What are the implications of the main findings?**

- Parental under-recognition of childhood overweight and obesity may delay early intervention, reducing opportunities for families and healthcare providers to engage in preventive behaviours and support healthy growth before obesity-related health risks develop;
- Health promotion initiatives should be culturally safe, community-informed, and grounded in Indigenous perspectives of health, recognizing that parental perceptions of child weight are influenced by physical activity, community norms, historical experiences, and broader social determinants of health rather than simply a lack of knowledge.

**Abstract:**

Background/Objectives: Indigenous children in Canada face higher rates of overweight and obesity compared to non-Indigenous children. Parents’ perceptions of their child’s body weight are shaped by broader systemic influences, including historical trauma and limited access to healthy food. This study aimed to evaluate the association between children’s actual body weight (measured by BMI) and parental perceptions among First Nations children. Methods: Participants were children from two Saskatchewan First Nations communities involved in the First Nations Lung Health Project (2013 and 2017). Self-administered questionnaires were distributed through schools to parents of children in Grades 1–12. The questionnaire collected data on health, environment, physical activity, and diet. Parents assessed their child’s weight as underweight, about the right weight, or overweight. BMI was calculated using clinically measured height and weight. Logistic regression was used to identify predictors of BMI misclassification. Results: Among 472 children with both survey and clinical data, 42.6% were boys. Clinically measured overweight prevalence was 52.3%, compared to 15.9% based on parental perception. Overall, 58% of parents accurately perceived their child’s weight status. Misclassification was more likely among children who engaged in at least 60 min of daily physical activity on more days per week. Conclusions: Parents underestimated their children’s overweight status compared to clinical measures. Questionnaire-based assessments significantly underestimated true weight status among school-aged First Nations children. These findings highlight the need to consider systemic and environmental factors when addressing childhood obesity in First Nations communities.

## 1. Introduction

Childhood obesity has emerged as one of the most persistent and complex public health challenges in Canada, mirroring global trends and reflecting decades of gradual increases in excess weight among children and adolescents. Childhood obesity carries substantial long-term health consequences. According to Public Health Agency of Canada’s (PHAC’s) national report, high BMI in childhood is a significant predictor of adult obesity, which is associated with chronic diseases such as type 2 diabetes, cardiovascular disease, and certain cancers. Psychological effects including depression, low self-esteem, and behavioural difficulties are also commonly observed among children with obesity [1].

National surveillance systems, including the Canadian Health Measures Survey (CHMS) and reports from the PHAC, demonstrate that despite sustained prevention efforts, obesity prevalence among Canadian youth remains high and shows little indication of meaningful decline. In 2015, 12.4% of Canadian children and adolescents aged 5 to 17 years had a body mass index (BMI) that classified them as obese, while an additional 19.4% were classified as overweight. Consequently, nearly one-third (31.8%) of Canadian children and adolescents fell within the combined overweight and obesity category [1]. The prevalence of obesity was higher among boys than girls (14.9% vs. 9.9%). Similarly, the prevalence of combined overweight and obesity was greater among adolescents aged 12 to 17 years compared with children aged 5 to 11 years (35.6% vs. 28.4%) [1].

Indigenous (First Nations, Inuit, and Métis) children have higher rates of overweight and obesity than non-Indigenous children in Canada. This disparity is well-documented in public health literature and is attributed to food insecurity, colonial impacts, and social determinants of health [2]. According to the First Nations Regional Longitudinal Health Survey (RHS) data on First Nations on-reserve and northern communities, 22.3% of First Nations children (0–11 years) are overweight and 36.2% of First Nations children are obese [3]. This means that over half (approximately 58%) of First Nations children fall into a category of overweight or obesity. The majority of youth (aged 12 to 17 years) are normal/underweight, but 28% of First Nations adolescents are considered overweight and 14% are classified as obese [3].

One of the most significant contributors to obesity among Indigenous children is food insecurity, which remains disproportionately high in First Nations, Inuit, and Métis communities. A 2023 review published in PLoS Global Public Health identifies food insecurity as being at crisis levels in many Indigenous communities, with lasting effects on children’s nutritional health, cognitive development, and long-term wellbeing. The review finds that the high cost of food relative to income, especially in remote northern communities, severely limits access to healthy foods and forces reliance on high-calorie, low-nutrient market foods, contributing to obesity risk among Indigenous children [4].

This issue is deeply rooted in historical and structural inequities. Indigenous-focused research highlights that obesity among First Nations, Inuit, and Métis children cannot be fully understood without acknowledging the ongoing impacts of colonization. Colonial practices, including the forced displacement of Indigenous peoples from their traditional lands, the disruption of traditional food systems, and restrictive government policies, have significantly affected communities’ ability to access and maintain traditional diets and food practices. Historically, traditional foods provided essential nutrients and supported active, healthy lifestyles. The loss of access to these foods has contributed to increased reliance on less nutritious market foods, resulting in a complex health landscape characterized by both nutritional deficiencies and rising rates of obesity. This phenomenon reflects a “double burden” of malnutrition, where undernutrition and obesity coexist within the same populations and communities [5,6,7].

A growing body of recent research also confirms that parental misperception of child weight status remains widespread [8,9,10,11,12,13,14,15,16,17,18,19]. PHAC’s 2024 national obesity report emphasizes the continued limitations of self- and parent-reported anthropometric data and the need for clinically measured indicators to ensure accuracy, underscoring the discrepancy between reported and measured BMI among Canadian children [1]. Parental misperception of child weight status may contribute to childhood obesity among Indigenous populations as well; however, the available literature on this issue remains limited. Studies have shown that parents often underestimate or fail to recognize overweight and obesity in their children, which can reduce the likelihood of early intervention, healthy lifestyle modifications, or seeking professional support [2,7]. In Indigenous communities, perceptions of healthy body size may be influenced by cultural norms, historical experiences of food insecurity, and broader social determinants of health. These factors can make the identification of unhealthy weight gain more challenging and may contribute to the persistence of childhood obesity. Furthermore, high rates of obesity and overweight within some communities may normalize larger body sizes, leading parents to perceive excess weight as typical or healthy [2,20]. Understanding parental perceptions is therefore an important component of culturally appropriate obesity prevention and intervention strategies for First Nations, Inuit, and Métis children [21,22].

Parental perception remains a critical factor, as accurate recognition of excess weight is strongly linked to engagement in preventive strategies such as promoting healthy dietary behaviours and physical activity. Despite the importance of this issue, only a limited number of Canadian studies have directly compared parental perceptions with clinically measured BMI among children highlighting a key gap in current national research [2,16]. This topic remains understudied and there is not much information available for First Nations, Inuit, and Métis populations in Canada, and much of the available evidence on parental weight perception comes from Australian Indigenous populations and general childhood obesity research [22,23]. The primary objective of this paper was to study the child’s actual body weight and BMI compared to a parent’s perspective of weight status of a group of rural Saskatchewan First Nations children. Other objectives were to compare responses of questionnaire reported weight status, their perceptions of weight status and objectively measured weight status on BMI category scale and to identify predictors of inaccurate parental perception of their children’s weight status among school aged First Nations children.

## 2. Materials and Methods

### 2.1. Subjects and Procedure

The study population consisted of First Nations children living in two reserve communities in Saskatchewan. First Nations are one of the three Indigenous Peoples in Canada, alongside the Inuit and Métis. Each First Nation has distinct heritage, language, cultural practices, and spiritual beliefs [24,25]. The Government of Canada designated specific parcels of land, known as “reserves,” for the use and benefit of First Nations peoples, and treaty agreements outline mutual promises, responsibilities, and benefits between First Nations and the federal government [24].

This analysis used data from the child component of the baseline survey conducted as part of the First Nations Lung Health Project (FNLHP) [26]. The overall study design has been described elsewhere [27] and was conducted in accordance with the Canadian research ethics guidelines mentioned in the Tri-Council Policy Statement: Ethical Conduct for Research Involving Humans (TCPS2) [28]. Since the inception of the FNLHP, First Nations Elders have provided guidance and have been fully informed and supportive of the project. Community feedback was incorporated at every stage of the study to address emerging issues. The project manager met with Elders from both communities to review consent and assent forms as well as the questionnaire. In-person meetings with school staff (7 December 2012; 18 January 2013; and 7 February 2013) resulted in verbal support from all participating schools. Community members also contributed to the development of the child questionnaires.

Children enrolled in kindergarten through Grade 12 at four schools, including one school in Community A and three schools in Community B, were eligible to participate in the study. Approval to conduct the research was obtained from relevant authorities, including the school division superintendent, directors of education, and school principals. Data collection was carried out in 2013. A survey questionnaire was distributed to parents or caregivers through classroom teachers. The questionnaire collected information on sociodemographic characteristics, child health status, history of childhood and other illnesses, lifestyle factors, home environment, health risk behaviours, access to healthcare services, and family history of respiratory disease. Research nurses retrieved completed study packages from the schools two weeks later. Parents or caregivers received a CAD $5.00 gift card as an incentive for returning a completed questionnaire. Of the 603 survey packages distributed to parents or caregivers of children aged 6 to 17 years, 351 were returned, yielding a response rate of 58.2%. The study protocol was reviewed and approved by the University of Saskatchewan Biomedical Research Ethics Board (Bio #13-27) before data collection commenced.

The same procedures were followed for the 2017 follow-up survey (Phase 2). In 2017, 824 survey packages were distributed to parents of children aged 6–17 years, and 233 (28.3%) were returned. Of these, 47 students had also participated in the 2013 baseline survey and were excluded to avoid duplication. The final study sample comprised 537 children (351 from Phase 1 and 186 from Phase 2). Information for each child was provided by one parent (either the mother or father) or another caregiver. Clinical measurements of height and weight were available for 472 of these children, and the present analysis is based on this subset.

Children underwent clinical assessments of abdominal girth, height, and weight. Height was measured using a wall-mounted measuring tape, with children standing barefoot or in socks on a firm, level surface. Weight was measured using a calibrated spring scale while children wore light indoor clothing and socks. Body mass index (BMI) was calculated as weight in kilograms divided by height in metres squared (kg/m^2^), using the standard formula: BMI = weight (kg)/height (m)^2^ [29]. Additional data were collected on self-reported height and weight, dietary habits (including weekly frequency of food consumption), physical activity levels, home environment, parental education, and past and current health history of the child.

### 2.2. Parents’ Perception of Children’s Weight Status

Parents’ perceptions of their children’s weight were assessed using the question, “Do you consider your child to be underweight, just about the right weight, or overweight?” This item was adapted from a measure used in the National Health and Nutrition Examination Survey III (NHANES III) [30].

### 2.3. Classification of Overweight and Obesity

Clinically measured height and weight were used to calculate body mass index (BMI). Weight status categories were assigned according to age- and sex-specific BMI cut-off values established by the International Obesity Task Force (IOTF) [31,32]. The child’s objectively measured weight status (underweight, normal weight, or overweight) was compared with the parent’s reported perception of their child’s weight.

For the purpose of analysis, the IOTF-defined overweight and obese categories were combined into a single “overweight/obese” group. A binary variable was created to indicate whether parents accurately perceived their child’s BMI category. Misclassification was defined as any discrepancy between the child’s actual BMI category and parent’s perceived weight status. The binary variable was coded as “yes” if: (1) an overweight/obese child was perceived as normal weight or underweight; (2) a normal-weight child was perceived as underweight or overweight; and (3) an underweight child was perceived as normal weight or overweight. If there was no discrepancy between the child’s actual BMI category and the parent’s perceived weight status, the binary variable was coded as “no”. This binary misclassification variable (yes/no) served as the primary outcome in the logistic regression analysis. Although most misclassification occurred in the expected direction, typically underestimation of the child’s true weight status, instances of both underestimation and overestimation were examined and described.

### 2.4. Statistical Analysis

All statistical analyses were conducted using SPSS version 31 (IBM Corp., Armonk, NY, USA; 2025). Descriptive statistics, including frequencies, measures of central tendency and variability, and Chi-square tests, were used to summarize participant characteristics and assess bivariate associations. Logistic regression analysis was used to examine the association between BMI misclassification (yes/no) and potential explanatory variables. Variables with a *p*-value < 0.20 in bivariate analyses were considered candidates for inclusion in the multivariable model. The final model retained all statistically significant variables (*p* < 0.05) as well as conceptually important covariates. Model parsimony was assessed using the Hosmer–Lemeshow goodness-of-fit test [33]. Results are presented as odds ratios (ORs) with corresponding 95% confidence intervals (CIs).

## 3. Results

A total of 472 children participated in both the questionnaire survey and clinical assessments. Among them, 201 were boys (42.6%) and 271 were girls (57.4%). The mean age of the sample was approximately 10.5 years (SD = 3.1). According to parental perception, 15.9% of the children were considered overweight. However, clinical measurements indicated a substantially higher prevalence of overweight at 52.3% (Table 1).

Table 2 shows the association between parental perceptions and reports of their child’s weight status and the child’s objectively measured weight status during clinical assessment. The majority of parents (89.9%) correctly classified their child as being “just about the right weight.” However, among children classified as overweight based on clinical measures, 68.9% of parents still perceived their child as being of normal weight, indicating a substantial level of misperception among parents of overweight children.

When compared with the child’s actual measured weight, parental reports of weight status were more accurate than parental perceptions for identifying underweight and overweight children (underweight: 44.4% vs. 40.0%; overweight: 74.9% vs. 30.7%). In contrast, for children of normal weight, parental perception was more accurate than parental report in correctly identifying normal weight status (89.9% vs. 65.4%) (see Table 2).

Overall, the accuracy of parental perception of weight status in the total sample was 57.9%, whereas the accuracy of parent-reported weight status was higher at 69.9%. These findings indicate that parental reports of weight status were generally more accurate than parental perceptions.

Table 3 presents a comparison between parental reports and actual measurements of weight, height, and calculated BMI, stratified by sex. There was a significant underestimation of both weight and height for boys and girls. However, no significant differences were observed between parent-reported and clinically measured BMI in either sex.

**Figure 1:**
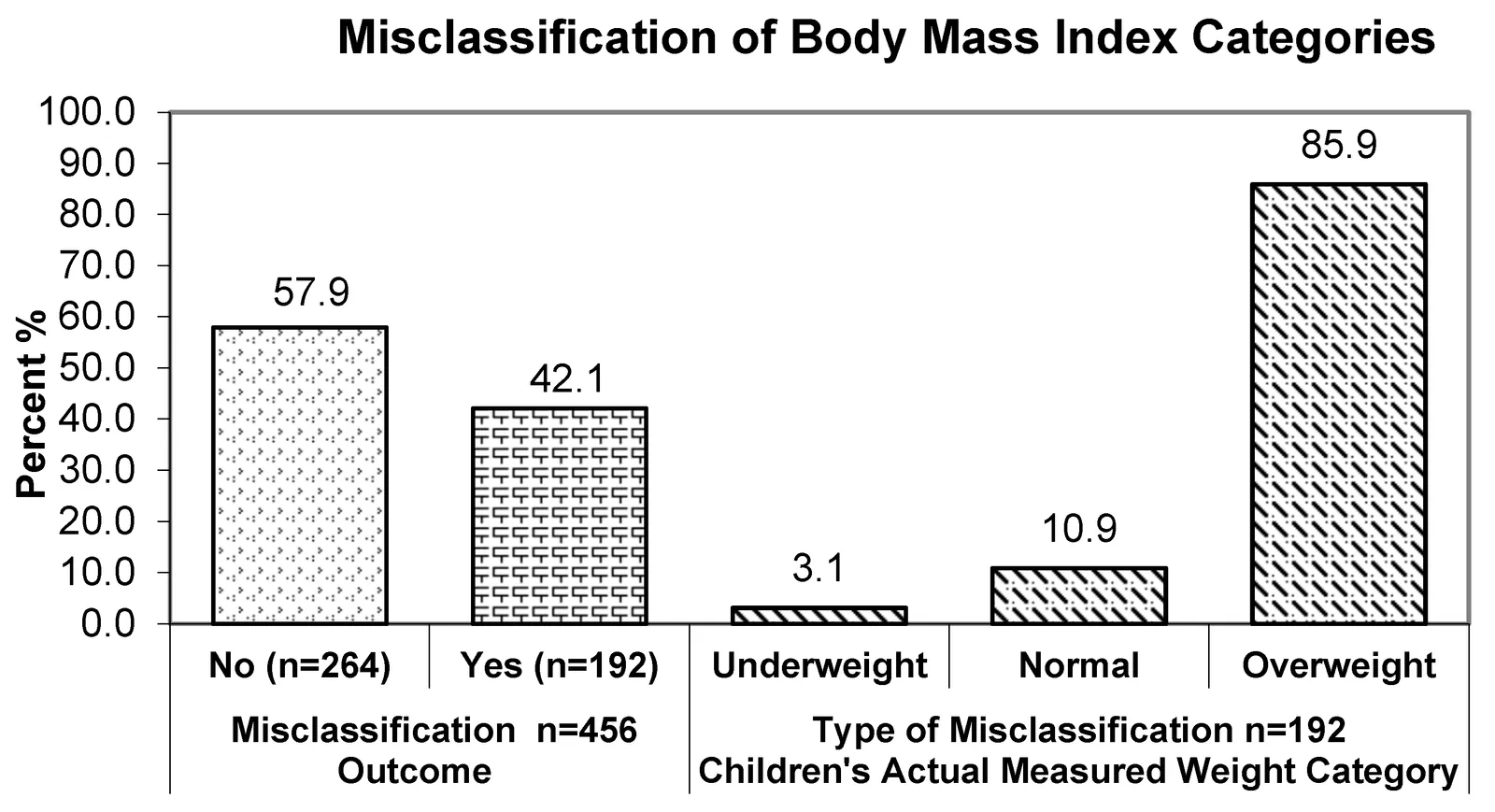
Misclassification of body mass index categories of children in two Saskatchewan First Nations Communities; for example, 85.9% actual overweight children were misclassified as underweight or normal weight by their parents, and 3.1% of actual underweight children were misclassified as normal weight. Outcome variable was defined as misclassified or not (Yes/No).

The misclassification analysis was restricted to 456 children with both clinical measurements and parental perception data available. Overall, 42.1% of children (192/456) were misclassified into underweight, normal weight, or overweight categories. Figure 1 illustrates the distribution of misclassification categories. Among the misclassified children, the majority (85.9%) belonged to the overweight group, followed by normal weight (10.9%) and underweight children (3.1%).

All but one parent of an overweight child who was misclassified underestimated their child’s weight status. In contrast, all parents of misclassified underweight children overestimated their child’s weight. Among parents of misclassified normal-weight children, most (90.5%) underestimated their child’s weight status, whereas only 9.5% overestimated it. These findings indicate that underestimation of child weight status was substantially more common than overestimation across parental misclassification groups.

Misclassification was slightly higher among boys (43.4%) than girls (41.2%). When stratified by sex, underestimation was also more common in boys (42.3%) compared to girls (38.8%); however, this difference was not statistically significant (*p* = 0.451).

Logistic regression analysis was conducted to identify predictors of misclassification between parents’ perceived child weight status and clinically measured BMI. Both unadjusted and adjusted results are presented in Table 4. After adjusting for covariates, the only significant predictor of misclassification was the number of days children engaged in at least 60 min of daily physical activity (OR = 1.13), indicating that higher levels of reported physical activity were associated with an increased likelihood of misclassification.

The Hosmer–Lemeshow goodness-of-fit test (χ^2^ = 8.60, df = 8; *p* = 0.377) indicated that the observed and predicted values were not significantly different, suggesting that the model demonstrated a good fit. No other variables showed a statistically significant association with misclassification of parental perception of child weight status (Table 4).

## 4. Discussion

This study demonstrates that accurately recognizing overweight or obesity in children is a significant challenge for First Nations parents. Parents were more likely to report their child’s weight accurately when providing height and weight measurements separately than when classifying their child into categories such as underweight, normal weight, or overweight. Overall, 42.1% of parents misclassified their child’s weight status. Among these misclassifications, 85.9% involved underestimation of overweight status. Furthermore, higher levels of child physical activity were associated with an increased likelihood of parental misclassification of weight status. Specifically, parents were more likely to misclassify their child’s weight when the child participated in at least 60 min of physical activity on more days per week.

The finding that greater physical activity was independently associated with parental misclassification of a child’s weight status is noteworthy. One possible explanation is that parents may perceive physical activity as a marker of good health and therefore be less likely to recognize excess weight in a child who is active. In other words, parents may equate an active lifestyle with a healthy weight, regardless of the child’s actual BMI classification. Physical activity may also make excess body weight less noticeable if children are perceived as fit, athletic, or generally healthy. Additionally, physical activity in this study was assessed through parental report and is therefore subject to recall and social desirability biases. Parents who overestimate their child’s activity levels may also be more likely to underestimate or misclassify their child’s weight status. Consequently, the observed association should be interpreted with caution, and future studies using objective measures of physical activity may help clarify the relationship between activity levels and parental perception of child weight status.

Although First Nations parents tended to underestimate their children’s weight, height, and BMI compared with clinically measured values, the mean differences were small in this study. Similar findings have been reported in previous studies. Scholtens et al. demonstrated a high level of agreement between parent-reported and objectively measured anthropometric data, suggesting that parents can generally provide reliable information regarding their child’s growth parameters [34]. Likewise, Powell-Young found that parents frequently underestimated their children’s weight and overestimated their height, resulting in the underestimation of BMI values [35]. Despite these biases, parent-reported measures remain a practical and valuable alternative in large-scale epidemiological studies where direct measurements are not feasible. Previous research suggests that parent-reported data can provide reasonably accurate population-level estimates, particularly in settings where objective measurements are logistically challenging [36,37,38].

Numerous studies have reported that parents often have difficulty accurately identifying their children’s weight status, with misclassification rates ranging from 20% to 80% [8,9,12,14,30,39,40,41,42,43,44,45,46,47,48,49,50]. This evidence suggests that parental misperception of child weight is a widespread issue across diverse populations and settings. For example, a Canadian study found that 63% of parents classified their overweight children as having a normal weight, highlighting a substantial tendency toward underestimation of excess weight among children [16]. These findings are consistent with the current study, in which parental underestimation was the predominant form of weight-status misclassification. Similarly, a systematic review reported that mothers frequently underestimate their children’s overweight and obesity status [51]. This pattern is also evident among First Nations and other Indigenous populations, where childhood obesity rates are disproportionately higher than among non-Indigenous children and closely associated with broader social determinants of health, including ongoing impacts of colonization, poverty, and inequitable access to resources [22,52].

Evidence suggests that parents have limited ability to identify overweight and obesity in their children when assessed against internationally recommended weight-status classification standards [41]. Among First Nations families, this may reflect differing cultural perspectives on body size and health, where weight is not always viewed in isolation but as one component of overall well-being, including physical, emotional, and cultural health. In some contexts, larger body size may not carry the same negative connotations and may be normalized within communities where overweight and obesity are prevalent. Structural factors, including the disproportionately high burden of food insecurity among Indigenous children may further influence how parents perceive and prioritize healthy weight [4,21,22].

In addition, Indigenous parents’ perceptions of their children’s weight status may be influenced by their own weight status as well as community norms and cultural beliefs regarding body size. As a result, larger body sizes may be viewed as healthy, normal, or desirable within some communities, which can contribute to the under recognition of overweight and obesity among children. Studies have found relatively low levels of parental concern about childhood overweight among Indigenous families, and research more broadly suggests that parents with overweight or obesity are more likely to underestimate their children’s weight status. This may contribute to delayed recognition of childhood overweight and reduced engagement in weight-management behaviours [22,23,53,54].

Parents may therefore place greater emphasis on functional measures of health such as physical activity and strength rather than on BMI classifications [49]. In addition, some parents may be less likely to rely on clinical indicators such as BMI percentiles or physician assessments, particularly given Indigenous peoples’ historical and ongoing distrust of health systems and the limitations of BMI as a culturally neutral measure of health [55]. Jones et al. noted that parents often rely on visual comparisons with other children when assessing weight status [41]; in communities where higher body weight is more prevalent, this relative comparison may further normalize overweight and contribute to its under-recognition.

Overall, these findings suggest that parental misclassification of child weight status in First Nations communities should be interpreted within a broader socio-cultural and structural context, rather than solely as a lack of knowledge, highlighting the need for culturally appropriate and community-informed approaches to addressing childhood obesity.

Socioeconomic inequalities play a major role in shaping childhood weight outcomes in Canada. National data show that children from lower-income households have significantly higher rates of overweight and obesity compared with those from higher-income households, reflecting disparities in access to healthy foods, safe recreational environments, and opportunities for physical activity. The Global Obesity Observatory further highlights pronounced differences by socioeconomic group, where childhood overweight and obesity are highest among those experiencing financial hardship and lowest in families with greater socioeconomic stability [56]. For Indigenous populations, socioeconomic determinants are magnified. Systemic barriers including poverty, inadequate housing, and underfunded community infrastructure contribute to environments that predispose children to unhealthy weight gain. These conditions intersect with cultural and historical factors (e.g., intergenerational trauma, disruption of traditional economies) to create layered vulnerabilities.

Indigenous-specific literature emphasizes that obesity among First Nations, Inuit, and Métis children cannot be fully understood without considering the impacts of colonization. The disruption of traditional food systems, residential school trauma, erosion of Indigenous languages, and systemic marginalization have resulted in intergenerational health consequences. The PLoS Global Public Health review identifies the transition from traditional foods to inexpensive, processed market foods as a direct consequence of socioeconomic constraints and colonization-driven food system disruptions, which in turn heightens obesity risk among Indigenous children [4]. Culturally appropriate interventions that incorporate Indigenous Knowledge, community leadership, and food sovereignty principles have been identified as essential to addressing obesity inequities.

In Canada, geographic disparities play a significant role in childhood obesity risk, particularly among children living in rural, northern, and remote regions where access to affordable nutritious foods, healthcare services, and recreational opportunities is limited [4,57]. The high cost of food and high rates of food insecurity in Inuit Nunangat and other northern communities contribute to poorer dietary quality and greater reliance on energy-dense processed foods [4,58]. Furthermore, climate change is disrupting traditional food systems by affecting access to country foods and altering environmental conditions essential for harvesting activities, thereby exacerbating nutritional inequities and obesity risk among Indigenous children [58,59].

Evidence on body weight perception indicates that weight perception is related to physical activity behaviour. Similar findings have been reported among Indigenous populations. In a study of overweight and obese American Indian and Alaska Native adolescents, those who misperceived their weight status were more likely to report engaging in at least 60 min of physical activity on five or more days per week than adolescents who accurately perceived themselves as overweight or obese [60]. Furthermore, adolescents who misperceived their weight status were less likely to engage in unhealthy fasting behaviors. These findings suggest that self-perceived weight status may influence health-related behaviours and should therefore be considered when designing weight management and obesity prevention interventions for Indigenous youth. Further research is needed to better understand the relationship between weight perception, physical activity, and weight-related behaviours in this population [60].

This study has several notable strengths. First, it had sufficient statistical power to adequately address the study objectives. It also examined a range of additional factors not commonly reported in similar studies, including household structure (single-parent vs. two-parent/partner households), physical activity levels, access to healthcare, fast food and beverage consumption, and the presence of health conditions. None of these factors showed a significant association with the misclassification of parental perception of a child’s weight status except the level of physical activity. A notable strength of this study was the inclusion of objective anthropometric measurements, which enabled validation of parental reports of their children’s weight and height and enhanced the accuracy of weight-status classification.

In this analysis, children’s weight status was classified using age- and sex-specific BMI categories in accordance with established reference standards [31,32]. BMI is a simple, cost-effective measure that adjusts weight for height and is widely used in both clinical and population health research [61,62]. It is a standardized and internationally accepted metric, allowing comparison across populations and over time [63]. Although BMI does not directly measure body fat, it has been shown to correlate reasonably well with adiposity and is strongly associated with obesity-related health risks, including cardiometabolic conditions [64,65,66]. In children, age- and sex-specific BMI percentiles account for growth and developmental differences, improving its applicability in pediatric populations [67]. However, the association between BMI and body fatness is affected by factors such as age, sex, height, and sexual maturation [68]. While age, sex, and height were considered in the present analysis, data on sexual maturation were not available, which may have influenced the interpretation of BMI-based weight classifications.

Although this study provides valuable insights into parental perceptions of child weight status, several limitations should be acknowledged. The sample was limited to two rural First Nations reserves in Saskatchewan, which may restrict the generalizability of the findings to other Indigenous communities and Canadian reserves. Despite this limitation, the results indicate that many parents had difficulty accurately identifying their children’s weight status when compared with BMI-based weight classifications. One possible explanation is a limited understanding of BMI cut-offs, particularly the distinction between healthy weight and overweight categories. Alternatively, parents may recognize their child’s weight status but be reluctant to classify their child as overweight because of social desirability or stigma associated with excess weight.

The response rate in the 2017 survey phase was 28.3%, which is comparable to many community-based surveys but may have introduced response bias. A comparison between participants with available anthropometric measurements and non-participants showed that participants were more likely to be female (57.4% vs. 36.9%; *p* = 0.002). However, there was no significant difference in age between the two groups (*p* = 0.359). Therefore, the findings may be subject to some degree of selection bias, particularly with respect to sex distribution.

We were unable to conduct sex-specific analyses to assess associations between covariates (e.g., physical activity levels) and weight status misclassification due to an insufficient number of misclassified cases among boys and girls. Additionally, physical activity was assessed based on parental self-reports. Although self-reported measures of physical activity are commonly used in epidemiological research, substantial discrepancies between self-reported and objectively measured activity levels have been well documented [69,70]. Such measures are subject to response bias, which may reduce the likelihood of detecting true associations between outcomes and behaviours such as physical activity or dietary intake [71,72]. This limitation may be particularly relevant in Indigenous contexts, where perceptions of healthy body size and weight may differ from standard biomedical definitions, and cultural norms can influence reporting and interpretation of health behaviours [73,74,75]. Previous research has shown that Indigenous parents may have different conceptualizations of child growth, body size, and health, which can contribute to variations in reporting and perception of weight status [7,74,75,76]. Although the use of objective measures of physical activity, such as accelerometry, and more precise dietary assessment methods, such as food diaries, would have strengthened the study, the additional costs and logistical requirements associated with these approaches were beyond the scope of the present study.

Another potential limitation was that the two recruitment periods may have differed with respect to background population characteristics and contextual factors. Temporal changes in obesity prevalence, evolving parental awareness of childhood weight and health behaviours, and the implementation of community or public health initiatives aimed at obesity prevention could have influenced participant characteristics and study outcomes. However, in the present study population, mean clinical BMI did not differ significantly between Period 1 (mean 22.30, SD 5.7, n = 308) and Period 2 (mean 22.12, SD 6.0, n = 164; *p* = 0.742). Similarly, the proportion of participants classified as overweight or obese was nearly identical in Period 1 (161/308, 52.3%) and Period 2 (86/164, 52.4%; *p* = 0.973). These findings suggest that the two cohorts were comparable with respect to BMI status, although the possibility of unmeasured temporal differences cannot be completely excluded.

An additional limitation is that participants were recruited from two Cree First Nations communities, and data from both communities were combined for analysis. Due to sample size limitations, community-specific analyses and adjustment for clustering by community were not conducted. Although both communities share a Cree cultural background, community-specific social, environmental, and historical contexts may influence perceptions of body size and health. As a result, combining data across communities may have masked potential differences between communities. Future research with larger samples should examine community-level variation and account for clustering by community where appropriate.

Furthermore, the cross-sectional design of this study precludes causal inference, and the direction of the observed association cannot be determined. Consequently, it is unclear whether greater physical activity influences parental perceptions of weight status, whether parental perceptions influence reports of physical activity, or whether both are related to other unmeasured factors. Future studies using longitudinal designs and objective measures of physical activity may help clarify these relationships.

These findings have important public health implications. Inaccurate parental perceptions of child weight status may delay the recognition of overweight and obesity and reduce engagement in preventive health behaviours. Therefore, interventions aimed at improving parental awareness and understanding of healthy child growth and weight status may be beneficial. Future research should examine the longitudinal consequences of parental misperceptions of children’s weight status and assess whether improving parental recognition contributes to healthier weight trajectories among Indigenous children.

## 5. Conclusions

In conclusion, the findings of this study indicate a high prevalence of overweight and obesity among First Nations children, highlighting an important public health concern within this population. Parents tended to underestimate their child’s overweight status when compared to clinically measured BMI. Questionnaire-based assessments of parental perception substantially underestimated true weight status among school-aged First Nations children. These findings highlight the need to address childhood obesity within the broader context of systemic, social, and environmental determinants that influence health outcomes in First Nations communities.

## Figures and Tables

**Table 1:** Characteristics of the study population, n = 472, children described using frequencies (%), means (SD: standard deviation) and medians (IQR: Inter quantile range).

Variable	Statistic
Age, in years (mean ± SD)	10.5 ± 3.1
Sex: n (%)	
Male	201 (42.6)
Female	271 (57.4)
Responder: n (%)	
Mother	309 (65.5)
Father	58 (12.3)
Caregiver	92 (19.5)
Missing	13 (2.8)
Responder’s highest education: n (%)	
Less than Grade 12	118 (25.0)
Completed Grade 12 or higher	298 (63.1)
Missing	56 (11.9)
Type of household: n (%)	
Single parent home	195 (41.3)
Two parents/partner home	261 (55.3)
Missing	16 (3.4)
Financial Strain-How much money left over at the end of the month: n (%)	
Some money	148 (31.4)
Just enough money	130 (27.5)
Not enough money	162 (34.3)
Missing	32 (6.8)
Ever experience any difficulties obtaining routine or on-going healthcare: n (%)	
Yes	30 (6.4)
No	416 (88.1)
Missing	26 (5.5)
During a normal week, number of days physically active at least 60 min:	
Mean ± SD	4.8 ± 2.1
Median (IQR)	5.0 (4.0)
During a normal week, number of hours per day child watch TV or play video games: n (%)	
Less than 1 h	54 (11.4)
1 h or more but less than 3 h	186 (39.4)
3 h or more but less than 5 h	137 (29.0)
5 h or more	88 (18.6)
Missing	7 (1.5)
Number of times per week, child eat chips, candy or drink pop: n (%)	
Less than or equal to 2 times	182 (38.6)
More than 2 times	262 (55.5)
Missing	28 (5.9)
Parent’s perception of child weight: n (%)	
Underweight	24 (5.1)
Just about the right weight	357 (75.6)
Overweight	75 (15.9)
Missing	16 (3.4)
Clinically measured BMI categories: n (%)	
Underweight	10 (2.1)
Normal	215 (45.6)
Overweight ^a^	247 (52.3)
Any health conditions ^b^ n (%)	
Yes	321 (68.0)
No	151 (32.0)

^a^ Overweight and obese categories combined. ^b^ Ever diagnosed with any health conditions includes wheeze, asthma, tonsillitis, bronchitis, pneumonia, eczema, croup, diabetes, ear infection, sleep apnea, heart conditions, whooping cough, and sinus.

**Table 2:** Proportion of parental perception of their child’s actual weight status and parental report of their child’s weight status (using height and weight) with actual measured weight status.

	Child’s Actual Measured Weight			Total n (%)	Test Statistic * and *p* Value
	Underweight n (%)	Normal n (%)	Overweight n (%)		
Parental Perceptions ^a^					N = 456; χ^2^ = 189.9; *p* < 0.001
Underweight	**4 (40.0)**	19 (9.1)	1 (0.4)	24 (5.3)	
Just about right weight	6 (60.0)	**187 (89.9)**	164 (68.9)	357 (78.3)	
Overweight	0 (0.0)	2 (1.0)	**73 (30.7)**	75 (16.4)	
Parental report of child’s weight ^b^					
Underweight	**4 (44.4)**	13 (10.0)	2 (1.2)	19 (6.2)	N = 306; χ^2^ = 308.6; *p* < 0.001
Just about right weight	4 (44.4)	**85 (65.4)**	40 (24.0)	129 (42.2)	
Overweight	1 (11.1)	32 (24.6)	**125 (74.9)**	158 (51.6)	

* Pearson Chi-squared test statistic and Fisher’s exact test *p* values were reported. Boldface values indicate accuracy in parent perception and parental report of child weight. ^a^ Accurate weight perception in total sample is 57.9% (=264/456). ^b^ Accurate weight reported in total sample is 69.9% (=214/306).

**Table 3:** Comparison of parent reported and actual clinical measured of weight, height, and BMI stratified by sex.

Variable	Boys Parent Reported Mean ± SE	Boys Clinical Mean ± SE	Girls Parent Reported Mean ± SE	Girls Clinical Mean ± SE
Weight in kg	49.2 ± 1.6	52.2 ± 1.9 *	47.9 ± 1.5	52.2 ± 1.6 *
Height in cm	147.3 ± 2.0	151.6 ± 1.5 *	146.3 ± 1.4	149.7 ± 1.1 *
Body mass index kg/m^2^	23.4 ± 0.65	22.5 ± 0.52	22.1 ± 0.5	22.7 ± 0.5

* *p* < 0.001; SE—Standard error of mean. Comparisons of parent reported and actual clinical measurements were made among boys and girls separately.

**Table 4:** Frequency of misclassification, unadjusted and adjusted odds ratios (ORs) and 95% confidence intervals (95% CI) based on logistic regression on misclassification of BMI; n = 456 ^a^.

Variable	Misclassification		Unadjusted OR (95% CI)	Adjusted OR (95% CI) ^c^
	Yes	No		
Age, in years (mean ± SD)	10.3 ± 3.1	10.6 ± 3.1	0.97 (0.92, 1.03)	0.99 (0.92, 1.08)
Sex: n (%)				
Male	85 (44.3)	111 (42.0)	1.00	1.00
Female	107 (55.7)	153 (58.0)	0.91 (0.62, 1.34)	1.07 (0.68, 1.69)
Responder: n (%)				
Mother	122 (65.2)	180 (69.8)	1.00	1.00
Father	29 (15.5)	25 (9.7)	1.71 (0.96, 3.06)	1.34 (0.69, 2.61)
Caregiver	36 (19.3)	53 (20.5)	1.00 (0.62, 1.62)	0.80 (0.43, 1.51)
Responder’s highest education: n (%)				
Less than Grade 12	53 (31.2)	64 (27.1)	1.22 (0.79, 1.88)	1.17 (0.71, 1.93)
Completed Grade 12 or higher	117 (68.8)	172 (72.9)	1.00	1.00
Type of household: n (%)				
Single parent home	78 (41.9)	110 (42.8)	0.96 (0.66, 1.41)	-
Two parents/partner home	108 (58.1)	147 (57.2)	1.00	
Financial Strain-How much money left over at the end of the month: n (%)				
Some money	59 (33.7)	87 (34.3)	1.00	-
Just enough money	56 (32.0)	70 (27.6)	1.18 (0.73, 1.91)	
Not enough money	60 (34.3)	97 (38.2)	0.91 (0.57, 1.45)	
Ever experience any difficulties obtaining routine or on-going healthcare: n (%)				
Yes	9 (5.0)	19 (7.5)	0.65 (0.29, 1.47)	0.55 (0.22, 1.39)
No	170 (95.0)	233 (92.5)	1.00	1.00
During a normal week, number of days physically active at least 60 min:				
Mean ± SD	5.1 ± 2.1	4.7 ± 2.2	1.09 (0.99, 1.20)	1.13 (1.01, 1.27)
During a normal week, number of hours per day child watch TV or play video games				
Less than 1 h	22 (11.6)	32 (12.2)	1.00	1.00
1 h or more but less than 3 h	77 (40.7)	100 (38.2)	1.12 (0.60, 2.08)	1.94 (0.86, 4.35)
3 h or more but less than 5 h	52 (27.5)	82 (31.3)	0.92 (0.48, 1.76)	1.66 (0.72, 3.86)
5 h or more	38 (20.1)	48 (18.3)	1.15 (0.58, 2.29)	2.44 (0.95, 6.29)
Number of times per week, child eat chips, candy, or drink pop				
Less than or equal to 2 times	80 (43.5)	95 (38.5)	1.00	1.00
More than 2 times	104 (56.5)	152 (61.5)	0.81 (0.55, 1.20)	0.65 (0.41, 1.03)
Any health conditions ^b^ n (%)				
Yes	134 (69.8)	181 (68.6)	1.06 (0.71, 1.59)	-
No	58 (30.2)	83 (31.4)	1.00	

^a^ For some variables numbers will not add up to 456 due to missing values. ^b^ Ever diagnosed with any health conditions includes wheeze, shortness of breath, asthma, hay fever, eczema, diabetes, arthritis, tonsillitis, ear infection, stomach reflex, and any allergy. ^c^ The multivariable logistic regression analysis was performed using complete-case analysis and included n = 349 observations.

## Data Availability

The First Nations community own and control the data and the data release as per the research agreements with the communities and the OCAP principles (https://fnigc.ca/ocap-training/) (accessed on 16 July 2024). Requests for data access can be made to the Chief and Council of the community A at reception@beardysband.com and to the Health Director of the community B at director@wchealth.ca.

## Abbreviations

The following abbreviations are used in this manuscript:

PHAC	Public Health Agency of Canada
CHMS	Canadian Health Measures Survey
RHS	First Nations Regional Health Survey
FNLHP	First Nations Lung Health Project
BMI	Body Mass Index
NHANES	National Health and Nutrition Examination Survey
IOTF	International Obesity Task Force
SPSS	Statistical Package for the Social Sciences
SD	Standard Deviation
OR	Odds Ratio
CI	Confidence Interval
